# Severe oropharyngeal dysphagia following COVID‐19: a case report

**DOI:** 10.1002/ccr3.3819

**Published:** 2021-01-28

**Authors:** Giulia De Vincentis, Chiara Ferrari, Dario Guerini Rocco

**Affiliations:** ^1^ Rehabilitation Unit ASST Bergamo Est Briolini Hospital Bergamo Italy

**Keywords:** COVID‐19, swallowing disorders, dysphagia rehabilitation, vocal fold paresis, case report

## Abstract

Dysphagia may occur after a prolonged intubation due to COVID‐19 but it is usually mild. Case reports on severe dysphagia following COVID‐19 are infrequent. Diagnosis can be difficult because international indications recommend avoiding instrumental assessments as far as possible because of the infection risk. An early rehabilitation treatment is recommended.

## Graphical abstract

This case report describes the clinical history of a COVID‐19 patient with a non‐immediate diagnosis of severe dysphagia that required a gastrostomy positioning. A vocal folds paresis was observed for the first time in the course of a Sars‐CoV‐2 infection. Thanks to intensive rehabilitation treatment, the outcome was favorable.

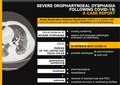

## 1 INTRODUCTION

Many patients who underwent prolonged intubation ^1^ develop dysphagia after extubation, with an occurrence varying between 3% and 62%. The prevalence of swallowing disorders increases as the intubation period is extended ^2^. Dysphagia predisposes to prolonged hospitalizations and to a worse prognosis due to the high risk of complications such as aspiration pneumonia, malnutrition, and dehydration.^1^ Laryngeal injury is a frequent consequence of intubation and only a small fraction of patients will emerge from intubation injury‐free.^3^ The origin of postintubation dysphagia derives from several factors. First of all, the endotracheal tube can cause trauma at pharynx or larynx with the onset of edema, mucosal abrasions, hematomas and ulcerations. Intubation may also result in dislocation and subluxation of the arytenoid cartilage and the endotracheal tube cuff can compress the branch of the recurrent laryngeal nerve. Moreover, complications in the intubation process or trauma are more frequent in emergency situation than in election surgery.^4^ Secondly, intubation can generate muscle atrophy due to disuse, aggravated by long sedation and the use of neuromuscular blockers. Such disuse of the swallowing mechanism may diminish its cortical representation and delay functional recovery in the long term. To these factors can be added changes in sensitivity, gastroesophageal reflux and breath‐swallowing incoordination.^3^ Recovery after postintubation dysphagia is usually possible in a short period of time.^2^ In patients with Acute Respiratory Distress Syndrome (ARDS), however, swallowing improvement appears slower, even longer than 6 months. The reason may lie in the severity of the pathology that often requires prolonged mechanical ventilation and long stays in intensive care unit.

A recent case‐control study compared the trend of swallowing disorders after endotracheal tube removal in 101 COVID‐19 patients and in 150 non‐COVID‐19 patients undergoing prolonged intubation. Although COVID‐19 patients were intubated longer, they showed less swallowing disorders after extubation and required fewer re‐education sessions.^5^ However, there are no data about the incidence of dysphagia in these patients also owing to the reduction of diagnostic procedures such as Fiberoptic Endoscopic Evaluation of Swallowing (FEES) during COVID‐19 pandemic because of the aerosol‐generating risk.^6^ In May 2020 the first case report on oropharyngeal dysphagia associated with Sars‐CoV‐2 was published: the patient, a 70‐year‐old man, after an 11‐day intubation developed dysphagia and a subsequent aspiration pneumonia. The instrumental evaluations evidenced bilateral absent GAG reflex, pyriform and vallecular salivary stagnation and silent aspiration. The patient received rehabilitative treatment with noticeable improvement of symptoms, without the need for nasogastric tube or Percutaneous Endoscopic Gastrostomy (PEG) positioning. The authors hypothesized the presence of a glossofaringeal and vagal neuropathy underlying the development of dysphagia.^7^ Another newest Italian case report highlighted the possible involvement of the cranial nerves in dysphagia development during COVID‐19.^8^

The purpose of the present case report is to describe the clinical history and the rehabilitation of a patient coming from Bergamo, Italy. Patient’s informed consent was obtained for data treatment.

## 2 CASE PRESENTATION

This patient, a 66‐year‐old man, developed a severe dysphagia of not immediate diagnosis during hospitalization for COVID‐19. He suffered from diabetes mellitus for about 10 years and chronic obstructive pulmonary disease (COPD). His drug therapy only consisted of metformin. He has never been hospitalized before. He accessed the Emergency Room on March 9, 2020, due to persistent fever for several weeks and worsening dyspnea. Nasopharyngeal swab for the detection of Sars‐CoV‐2 was positive. Chest computed tomography (CT) scan showed extensive inflammatory areas with ground‐glass opacities. Due to worsening respiratory pattern, the patient was placed on Continuous Positive Airway Pressure (CPAP) and started therapy with antibiotics, antivirals and hydroxychloroquine. In the following days, despite the use of CPAP, the clinical picture worsened up to ARDS and required tracheal intubation (on 12th March). On 24th March tracheotomy tube was placed. In the following weeks, there was a slow but progressive improvement in respiratory exchanges that allowed a gradual weaning from the ventilator. At the end of April a neurological evaluation was required due to loss of strength affecting the left lower limb. Brain CT showed no acute lesions. Because of abundant secretions in the airways, tracheal tube was not removed. Initial assessment of swallowing (nursing screening with Evan’s Blue dye test) performed at the beginning of May did not detect signs or symptoms of dysphagia, so parenteral nutrition was stopped and the patient started eating with modified diet and thickened liquids (honey‐thick and spoon‐thick). The texture of food was gradually modified from pureed diet to solid food. At the beginning of May nasopharyngeal swabs for detecting Sars‐CoV‐2 were negative and on May 22th the patient moved from the acute care hospital to the Rehabilitation Unit.

### 2.1 Differential diagnosis, investigations, and treatment

In the new hospital ward nursing screening test for dysphagia and specific evaluation of swallowing performed by speech and language pathologist (SLP) were carried out, as it is usually performed on tracheotomy patients at the beginning of the recovery. The tracheotomy tube was in place, the patient breathed in ambient air, using fenestrated inner cannula with decannulation plug. Evaluation of secretion management, Evan’s Blue dye test, test with gel‐water and methylene blue were negative. For preventive purposes, however, a pureed diet was recommended. In addition, evaluation during lunch was performed in order to detect any signs or symptoms of dysphagia: at the end of the meal some traces of bolus in the aspirate were found in association to wet voice. That same evening, while taking dinner with pureed diet under supervision, the presence of food material in the tracheal tube without reflex cough and desaturation (pulse oximeter readings from 99% to 94%) was observed. The meal was interrupted and the material was aspirated with rapid recovery of oxygen saturation. Subsequently, after the placement of the decannulation plug, wet voice was noticed. Since similar findings were also obtained by the SLP in the following days during daily evaluation at meal, oral feeding was interrupted due to the high risk of pulmonary complications. For this reason, on 25th May, after an unsuccessful attempt to place nasogastric tube, the medical team decided to feed the patient with parenteral nutrition. On 1st June gastroscopy was performed to exclude mechanical obstructions but it showed no notable alterations. On 3rd June the patient underwent FEES, which revealed a complete paresis of the right hemilarynx and a partial paresis of the left hemilarynx, in absence of heteroproductive lesions. The right paretic vocal fold was in paramedian position and the left vocal fold, with partial paresis, was in median position. There was sufficient breathing space. Swallowing was characterized by vallecular and retrocricoid salivary stagnation. A similar situation occurred during spoon‐thickened liquid swallowing test with stagnation and late inhalation. Reduced laryngeal sensibility and late and poor reflex cough were also observed. On 5th June the patient underwent head and neck CT scan to detect the possible causes of the vocal folds paresis. The CT scan showed a structural inhomogeneity just above the cricoarytenoid joints, sclerosis of the upper portion of the cricoid and of both arytenoids. The Radiologist reported that the alteration described was not purely characteristic for neoplasia, but more correlated to a chronic inflammatory event. For this reason, steroid therapy was introduced using intravenous methylprednisolone 20 mg twice a day for 15 days, followed by methylprednisolone 20 mg/day for a further 25 days. After FEES and CT, the medical team considered appropriate to position PEG (on 10th June). The patient underwent one‐hour speech therapy re‐education sessions from Monday to Friday, with the following objectives: prevention of arytenoid ankylosis, improvement of glottic closure and strengthening of the basis of the tongue. FEES, on 13th July, showed a clear improvement of glottic muscle: the right hemilarynx paresis remained but the left hemilarynx had a normal motility. Salivary stagnation was not present. Swallowing tests were normal with all consistencies. Therefore, it was indicated to start an oral nutrition re‐education, under supervision of a SLP. The patient started nutrition per os with pureed diet and spoon‐thickened liquid and constant training for compensatory posture under the SLP supervision. Daily calories requirement was guaranteed by lunch per os and two other meals by PEG. Administration of drug therapies was by PEG. The patient’s weight was measured daily. The oral intake was gradually increased and solid foods and liquids were introduced. On 16th July there was no more secretion or need to aspirate the patient, so tracheostomy tube was removed.

### 2.2 Outcome and follow‐up

**FIGURE 1:**
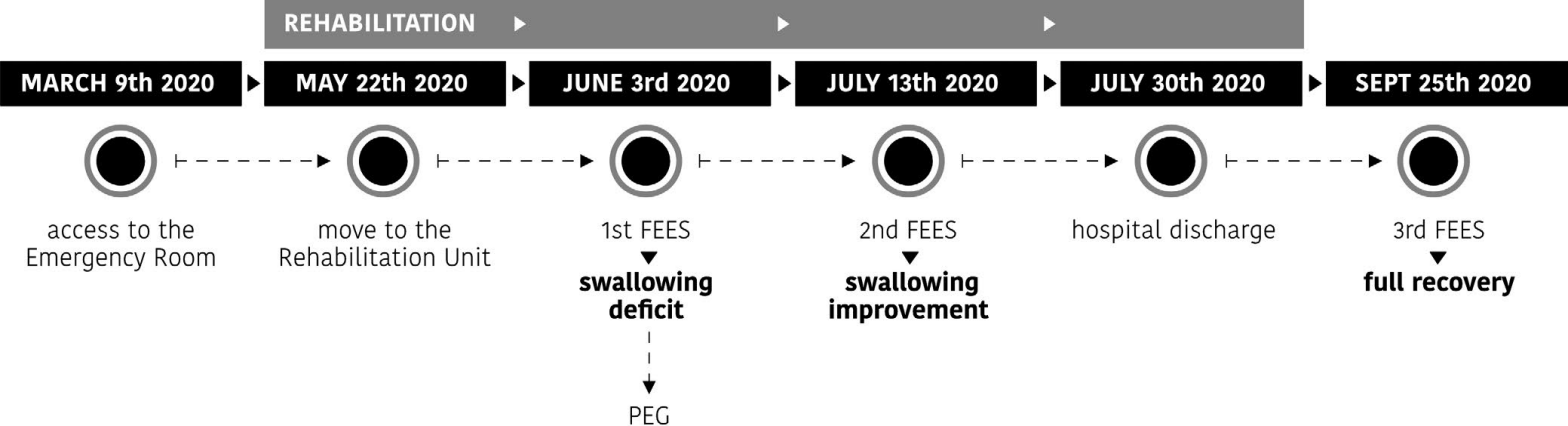
Timeline

On hospital discharge (30th July), the patient was fed with a diet without limitations in food consistencies and liquids with compensatory posture (head turned to the right). He took drug therapy orally. SLP gave guidance to perform independently daily exercises at home. PEG was no more necessary but the medical team decided to maintain the gastrostomic access until the Ear, Nose, and Throat (ENT) visit scheduled two months after the hospital discharge. On 25th September, the patient underwent ENT visit with FEES which showed no more paresis of the right hemilarynx and a full recovery. The ENT specialist allowed the PEG removal. The main steps of the patient’s course are summarized in the timeline (Figure 1).

## 3 DISCUSSION

Since the beginning of the 2020, about 130 others patients with COVID‐19 have been hospitalized in our Rehabilitation Unit. Among them, 17 were diagnosed with mild dysphagia, with a fast recovery. None of them needed enteral nutrition or prolonged speech therapy. Due to the severe respiratory failure, they also had a prolonged intubation, a tracheotomy (but for a maximum of one month) and were affected by a Critical Illness Myopathy and Neuropathy, but they did not develop a severe dysphagia. The patient of this case report was the only one who was still carrying the tracheotomy tube on his arrival and had to keep the tracheal tube because of abundant secretions in the airways, which were probably a result of many factors: the previous history of COPD, the recent pneumonia and the irritation of the airways due to inhalation.

This late inhalation was challenging to identify because the quality of the voice was already altered by the presence of the tracheotomy (despite the decannulation plug). Moreover, his voice was euphonic, not breathy, and he breathed well in ambient air with the decannulation plug. It is already known that vocal fold paresis is often misdiagnosed especially if the symptoms are nonspecific.^9^ For these reasons, it was difficult to guess a vocal fold paresis.

As it is documented in literature, swallowing with tracheotomy is more difficult due to reduced sensitivity stimulation, attenuated tracheal reflexes, inability to generate adequate under‐glottal pressure and reduced reflex cough. However, the presence of the tracheal tube has allowed us to directly establish inhalation since during these episodes there was not reflex cough as a result of a reduced laryngeal sensibility. These issues were confirmed by FEES, which was performed after observing the patient's performance at meal for several days. According to the ESSD commentary, the instrumental assessment has not been previously performed because of high infection risk.^6^

## 4 CONCLUSIONS

In summary, in this patient dysphagia lasted longer than expected after an intubation but the source seems neither the intubation nor the Sars‐CoV‐2 but an interaction of both. Investigating the possible etiology of vocal fold paresis, the instrumental examinations allowed us to exclude vascular events, neoplasms and degenerative diseases.^9^ It has not been possible to exclude postintubation, postviral and idiopathic etiology. With regard to the traumatic etiology linked to the intubation procedure, it must be considered that the urgent intubation procedure as practiced in our patient, compared to the elective one, exposes the patient to a higher risk of injury, with rates of up to 30% according to the literature.^4^ Concerning viral etiology, in literature there are studies that correlate respiratory viral infections to vocal fold paresis due to the virus neurotropism ^10^ and there is an increasing evidence that the nervous system is frequently involved in patients with COVID‐19.^11^ It was therefore not possible to identify with certainty the origin of vocal fold paresis in our patient.

Relating to the prognosis, there are no specific studies on recovery but the data suggest a good recovery within a year. Furthermore, this patient benefited from corticosteroid treatment, which targeted the hyperinflammation caused by the virus,^11^ and from intensive rehabilitation therapy with a good result in approximately 50 days.

At present, in literature there are not many studies about COVID‐19 and severe and prolonged dysphagia with the need of gastrostomy positioning. Moreover, vocal fold paresis during COVID‐19 was not described yet and for this reasons a comparison is difficult.

The present case report shows that dysphagia in COVID‐19 is not always mild and may cause serious complications. Therefore, we advise not to rely on a single evaluation and screening tests alone, but to monitor meal performance, especially in patients at high risk of dysphagia (such as those with long intubation or tracheotomy). Considering that sometimes the diagnosis of dysphagia is not obvious and the gold standard instrumental evaluations are limited due to the high risk of aerosol‐generating, awareness about dysphagia in this patient population should be increased. Moreover, it seems important to start an appropriate and intensive rehabilitation treatment early with high‐specialized professionals. We hope that the history of this patient will help other healthcare professionals during their clinical practice.

## CONFLICT OF INTEREST

The authors certify that there is no conflict of interest with any financial organization regarding the material discussed in the manuscript.

## AUTHOR CONTRIBUTIONS

GD and CF: acquired and analyzed the data, drafted the manuscript. DGR: revised critically the manuscript and supervised the project.

## ETHICS STATEMENT

Written informed consent was obtained from the patient for publication of this case report.

## Data Availability

No new data are presented in this report. Therefore, no data were deposited in data repositories.
